# 2-Carbamoyl-3,4,5,6-tetra­fluoro­benzoic acid

**DOI:** 10.1107/S1600536812036549

**Published:** 2012-09-05

**Authors:** Tao Lu, Xiao-Jian Liao, Yuan-Wei Liang, Shi-Hai Xu

**Affiliations:** a Department of Chemistry, Jinan University, Guangzhou 510632, People’s Republic of China

## Abstract

In the title compound, C_8_H_3_F_4_NO_3_, the carb­oxy group lies nearly in the plane of the ring with a C—C—C—O torsion angle of −10.5 (4)°. The carbamoyl group is almost perpendic­ular to the benzene ring [C—C—C—O torsion angle = 82.2 (4) °]. In the crystal, molecules are linked *via* O—H⋯O and N—H⋯O hydrogen bonds involving the carbamoyl and carb­oxy groups.

## Related literature

For general background to the title compound and its preparation, see: Xu *et al.* (2008 ▶); Li *et al.* (1999 ▶); Poshkus & Herweh (1957 ▶); Cai *et al.* (1992 ▶); Lee *et al.* (2005 ▶); Guo *et al.* (2011 ▶); Liao *et al.* (2011 ▶).
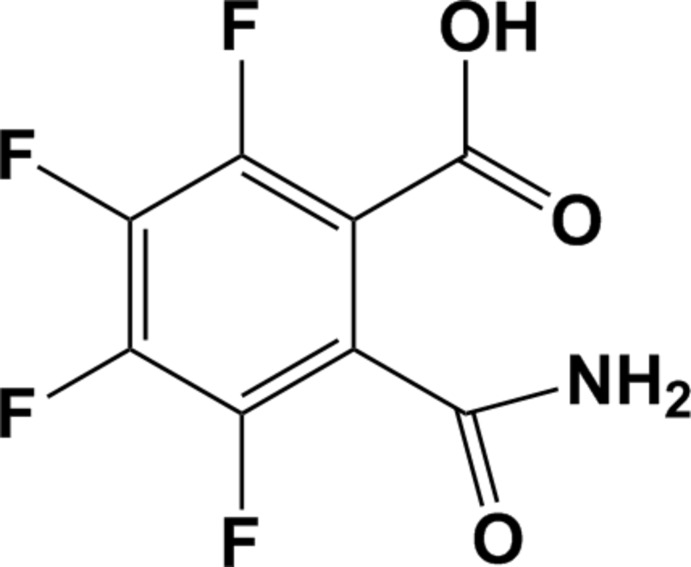


## Experimental

### 

#### Crystal data

C_8_H_3_F_4_NO_3_
*M* *_r_* = 237.11
Monoclinic, 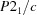

*a* = 14.5872 (6) Å
*b* = 6.9193 (3) Å
*c* = 8.6129 (4) Å
β = 100.086 (4)°
*V* = 855.89 (7) Å^3^
*Z* = 4
Cu *K*α radiation
μ = 1.78 mm^−1^
*T* = 268 K
0.42 × 0.31 × 0.17 mm

#### Data collection

Agilent Xcalibur Gemini ultra Sapphire-3 CCD diffractometer
Absorption correction: multi-scan (*CrysAlis PRO*; Agilent, 2011 ▶) *T*_min_ = 0.643, *T*_max_ = 1.000
2346 measured reflections
1312 independent reflections
1240 reflections with *I* > 2σ(*I*)
*R*_int_ = 0.013

#### Refinement

*R*[*F* ^2^ > 2σ(*F* ^2^)] = 0.035
*wR*(*F* ^2^) = 0.091
*S* = 1.14
1312 reflections
146 parameters
H-atom parameters constrained
Δρ_max_ = 0.23 e Å^−3^
Δρ_min_ = −0.17 e Å^−3^

### 

Data collection: *CrysAlis PRO* (Agilent, 2011 ▶); cell refinement: *CrysAlis PRO*; data reduction: *CrysAlis PRO*; program(s) used to solve structure: *SHELXS97* (Sheldrick, 2008 ▶); program(s) used to refine structure: *SHELXL97* (Sheldrick, 2008 ▶); molecular graphics: *OLEX2* (Dolomanov *et al.*, 2009 ▶); software used to prepare material for publication: *OLEX2*.

## Supplementary Material

Crystal structure: contains datablock(s) I, global. DOI: 10.1107/S1600536812036549/rk2367sup1.cif

Structure factors: contains datablock(s) I. DOI: 10.1107/S1600536812036549/rk2367Isup2.hkl

Additional supplementary materials:  crystallographic information; 3D view; checkCIF report

## Figures and Tables

**Table 1:** Hydrogen-bond geometry (Å, °)

*D*—H⋯*A*	*D*—H	H⋯*A*	*D*⋯*A*	*D*—H⋯*A*
O1—H1⋯O3^i^	0.82	1.78	2.5965 (18)	172
N1—H1*A*⋯O2^ii^	0.86	2.11	2.940 (2)	161

Symmetry codes: (i) 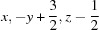; (ii) 
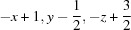
.

**Fig. 1.**
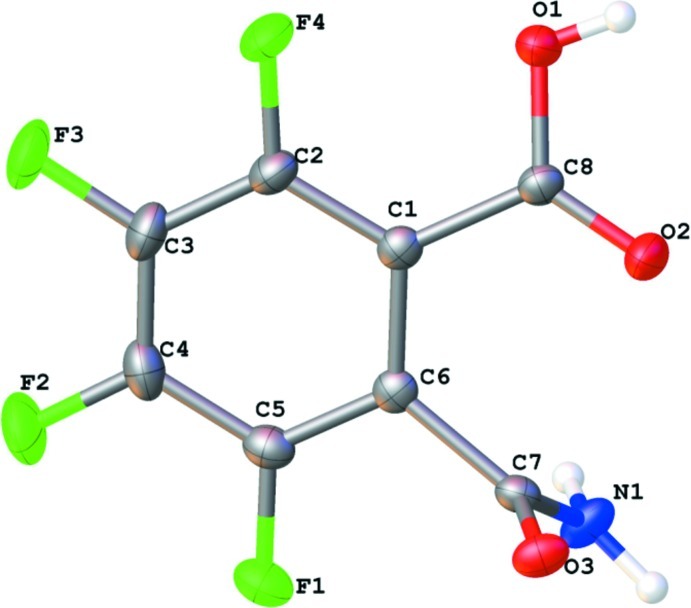
The molecular structure of the title compound with the atom numbering scheme. Displacement ellipsoids are drawn at the 50% probability level. H atoms are presented as a small spheres of arbitrary radius.

## supplementary crystallographic information

### Comment

The title compound, C_8_H_3_F_4_NO_3_ (Scheme 1, Fig. 1), as an intermediate in the synthesis of coupling reagent (Xu *et al.*, 2008; Li *et al.*, 1999; Poshkus & Herweh, 1957), was prepared by the reaction of 3,4,5,6-tetrafluorophthalic anhydride and ammonia gas at 268 K (Cai *et al.*, 1992; Lee *et al.*, 2005; Guo *et al.*, 2011; Liao *et al.*, 2011) with yield about 90%. The bond lengths and angles in the title molecule are unexceptional. The carboxyl group lies nearly in the plane of phenyl ring - torsion angle C6/C1/C8/O2 = -10.5 (4)°. The aminoacyl group is practically perpendicular to phenyl ring - torsion angle C1/C6/C7/O3 = 82.2 (4)°. In the crystal structure there are some intermolecular hydrogen bonds between the carbonyl- and amino-groups (Table 1) which form a network.

### Experimental

The 44.0 g 3,4,5,6-tetrafluorophthalic anhydride and 700 ml tetrahydrofuran were mixed to form a solution. After stirred solution was cooled to 268 K, ammonia gas was added at the pressure of 0.1 MPa for 1.5 h formed white solid. The white solid residue was dissolved in 350 ml distilled water, tetrahydrofuran was removed in vacuum at room temperature. Hydrochloric acid (2*N*) was added to adjust solution pH1 at 268 K, after the resulting the title product was obtained by filtration. The crude product was recrystallized from distilled water and methanol to give colourless crystals of the title product.

### Refinement

All H atoms were positioned geometrically and were included in the refinement in the riding-model approximation, with distances: 0.86Å (NH_2_), 0.82Å (OH) with *U*_iso_(H) = 1.2*U*_eq_(N) and *U*_iso_(H) = 1.5*U*_eq_(O) .

### Crystal data

**Table:**

C_8_H_3_F_4_NO_3_	*F*(000) = 472
*M**_r_* = 237.11	*D*_x_ = 1.840 Mg m^−^^3^
Monoclinic, *P*2_1_/*c*	Cu *K*α radiation, λ = 1.5418 Å
*a* = 14.5872 (6) Å	Cell parameters from 1702 reflections
*b* = 6.9193 (3) Å	θ = 5.2–62.6°
*c* = 8.6129 (4) Å	µ = 1.78 mm^−^^1^
β = 100.086 (4)°	*T* = 268 K
*V* = 855.89 (7) Å^3^	Block, colourless
*Z* = 4	0.42 × 0.31 × 0.17 mm

### Data collection

**Table:**

Agilent Xcalibur Gemini ultra Sapphire-3 CCD diffractometer	1312 independent reflections
Radiation source: Enhance Ultra (Cu) X-ray Source	1240 reflections with *I* > 2σ(*I*)
Mirror monochromator	*R*_int_ = 0.013
Detector resolution: 16.0288 pixels mm^-1^	θ_max_ = 62.7°, θ_min_ = 7.1°
ω scans	*h* = −15→16
Absorption correction: multi-scan (*CrysAlis PRO*; Agilent, 2011)	*k* = −4→7
*T*_min_ = 0.643, *T*_max_ = 1.000	*l* = −9→9
2346 measured reflections	

### Refinement

**Table:**

Refinement on *F*^2^	Primary atom site location: structure-invariant direct methods
Least-squares matrix: full	Secondary atom site location: difference Fourier map
*R*[*F*^2^ > 2σ(*F*^2^)] = 0.035	Hydrogen site location: inferred from neighbouring sites
*wR*(*F*^2^) = 0.091	H-atom parameters constrained
*S* = 1.14	*w* = 1/[σ^2^(*F*_o_^2^) + (0.0432*P*)^2^ + 0.5536*P*] where *P* = (*F*_o_^2^ + 2*F*_c_^2^)/3
1312 reflections	(Δ/σ)_max_ < 0.001
146 parameters	Δρ_max_ = 0.23 e Å^−^^3^
0 restraints	Δρ_min_ = −0.17 e Å^−^^3^

### Special details

**Table:**

Experimental. Absorption correction: *CrysAlisPro* (Agilent Technologies, 2011); Empirical absorption correction using spherical harmonics, implemented in SCALE3 ABSPACK scaling algorithm.
Geometry. All s.u.'s (except the s.u. in the dihedral angle between two l.s. planes) are estimated using the full covariance matrix. The cell s.u.'s are taken into account individually in the estimation of s.u.'s in distances, angles and torsion angles; correlations between s.u.'s in cell parameters are only used when they are defined by crystal symmetry. An approximate (isotropic) treatment of cell s.u.'s is used for estimating s.u.'s involving l.s. planes.
Refinement. Refinement of *F*^2^ against ALL reflections. The weighted *R*-factor *wR* and goodness of fit *S* are based on *F*^2^, conventional *R*-factors *R* are based on *F*, with *F* set to zero for negative *F*^2^. The threshold expression of *F*^2^ > σ(*F*^2^) is used only for calculating *R*-factors(gt) *etc*. and is not relevant to the choice of reflections for refinement. *R*-factors based on *F*^2^ are statistically about twice as large as those based on *F*, and *R*-factors based on ALL data will be even larger.

### Fractional atomic coordinates and isotropic or equivalent isotropic displacement parameters (Å^2^)

**Table:**

	*x*	*y*	*z*	*U*_iso_*/*U*_eq_	
F1	0.27285 (8)	0.04074 (16)	0.69216 (13)	0.0293 (3)	
F4	0.11543 (8)	0.66145 (18)	0.36824 (14)	0.0328 (3)	
F2	0.09507 (9)	0.02888 (19)	0.54379 (17)	0.0416 (4)	
O3	0.39455 (9)	0.41810 (19)	0.84775 (14)	0.0219 (3)	
F3	0.01714 (8)	0.3444 (2)	0.38240 (16)	0.0394 (4)	
O1	0.27710 (9)	0.8186 (2)	0.40108 (15)	0.0253 (4)	
H1	0.3157	0.9023	0.3929	0.038*	
O2	0.39129 (9)	0.7021 (2)	0.58320 (16)	0.0280 (4)	
N1	0.45248 (11)	0.2650 (2)	0.65574 (18)	0.0234 (4)	
H1A	0.5059	0.2493	0.7150	0.028*	
H1B	0.4431	0.2227	0.5604	0.028*	
C1	0.25134 (12)	0.5238 (3)	0.5229 (2)	0.0182 (4)	
C5	0.23637 (13)	0.1978 (3)	0.6119 (2)	0.0213 (4)	
C6	0.29012 (12)	0.3598 (3)	0.6086 (2)	0.0173 (4)	
C4	0.14478 (14)	0.1886 (3)	0.5367 (2)	0.0266 (5)	
C3	0.10574 (13)	0.3486 (3)	0.4559 (2)	0.0259 (5)	
C8	0.31341 (12)	0.6916 (3)	0.5074 (2)	0.0182 (4)	
C2	0.15862 (13)	0.5134 (3)	0.4498 (2)	0.0223 (4)	
C7	0.38555 (12)	0.3533 (3)	0.7106 (2)	0.0174 (4)	

### Atomic displacement parameters (Å^2^)

**Table:**

	*U*^11^	*U*^22^	*U*^33^	*U*^12^	*U*^13^	*U*^23^
F1	0.0368 (7)	0.0204 (6)	0.0312 (6)	0.0008 (5)	0.0074 (5)	0.0066 (5)
F4	0.0216 (6)	0.0325 (7)	0.0399 (7)	0.0031 (5)	−0.0069 (5)	0.0090 (5)
F2	0.0326 (7)	0.0308 (7)	0.0608 (9)	−0.0166 (6)	0.0066 (6)	0.0017 (6)
O3	0.0257 (7)	0.0245 (7)	0.0145 (7)	0.0056 (6)	0.0007 (5)	−0.0019 (5)
F3	0.0163 (6)	0.0463 (8)	0.0514 (8)	−0.0076 (5)	−0.0056 (5)	−0.0016 (6)
O1	0.0235 (7)	0.0248 (8)	0.0254 (7)	−0.0043 (6)	−0.0017 (6)	0.0084 (6)
O2	0.0205 (7)	0.0282 (8)	0.0318 (8)	−0.0053 (6)	−0.0053 (6)	0.0070 (6)
N1	0.0191 (8)	0.0329 (10)	0.0168 (8)	0.0069 (7)	−0.0009 (6)	−0.0051 (7)
C1	0.0191 (9)	0.0204 (10)	0.0153 (9)	0.0009 (7)	0.0036 (7)	−0.0015 (7)
C5	0.0257 (10)	0.0190 (10)	0.0203 (9)	0.0030 (8)	0.0068 (8)	0.0016 (7)
C6	0.0186 (9)	0.0200 (10)	0.0140 (8)	0.0013 (7)	0.0052 (7)	−0.0020 (7)
C4	0.0237 (10)	0.0265 (11)	0.0312 (11)	−0.0084 (8)	0.0090 (8)	−0.0043 (9)
C3	0.0153 (9)	0.0326 (12)	0.0288 (10)	−0.0020 (8)	0.0013 (8)	−0.0046 (9)
C8	0.0191 (9)	0.0198 (10)	0.0150 (9)	0.0012 (7)	0.0015 (7)	−0.0004 (7)
C2	0.0187 (9)	0.0262 (11)	0.0208 (9)	0.0036 (8)	0.0004 (7)	0.0014 (8)
C7	0.0213 (9)	0.0157 (9)	0.0153 (9)	0.0004 (7)	0.0032 (7)	0.0023 (7)

### Geometric parameters (Å, º)

**Table:**

F1—C5	1.346 (2)	N1—C7	1.308 (2)
F4—C2	1.336 (2)	C1—C6	1.416 (3)
F2—C4	1.329 (2)	C1—C8	1.493 (3)
O3—C7	1.249 (2)	C1—C2	1.390 (3)
F3—C3	1.336 (2)	C5—C6	1.371 (3)
O1—H1	0.8200	C5—C4	1.380 (3)
O1—C8	1.312 (2)	C6—C7	1.511 (2)
O2—C8	1.209 (2)	C4—C3	1.377 (3)
N1—H1A	0.8600	C3—C2	1.383 (3)
N1—H1B	0.8600		
			
C8—O1—H1	109.5	F2—C4—C3	120.74 (17)
H1A—N1—H1B	120.0	C3—C4—C5	118.76 (18)
C7—N1—H1A	120.0	F3—C3—C4	120.14 (18)
C7—N1—H1B	120.0	F3—C3—C2	119.97 (18)
C6—C1—C8	118.48 (16)	C4—C3—C2	119.88 (17)
C2—C1—C6	117.56 (17)	O1—C8—C1	113.98 (15)
C2—C1—C8	123.83 (17)	O2—C8—O1	124.27 (17)
F1—C5—C6	119.64 (16)	O2—C8—C1	121.69 (16)
F1—C5—C4	117.84 (17)	F4—C2—C1	122.02 (17)
C6—C5—C4	122.52 (18)	F4—C2—C3	115.95 (16)
C1—C6—C7	124.76 (16)	C3—C2—C1	122.02 (18)
C5—C6—C1	119.22 (16)	O3—C7—N1	123.22 (17)
C5—C6—C7	115.81 (16)	O3—C7—C6	118.21 (15)
F2—C4—C5	120.50 (18)	N1—C7—C6	118.29 (15)

### Hydrogen-bond geometry (Å, º)

**Table:**

*D*—H···*A*	*D*—H	H···*A*	*D*···*A*	*D*—H···*A*
O1—H1···O3^i^	0.82	1.78	2.5965 (18)	172
N1—H1*A*···O3^ii^	0.86	2.82	3.281 (2)	116
N1—H1*A*···O2^ii^	0.86	2.11	2.940 (2)	161

Symmetry codes: (i) *x*, −*y*+3/2, *z*−1/2; (ii) −*x*+1, *y*−1/2, −*z*+3/2.
